# May critical molecular cross-talk between indoleamine 2,3-dioxygenase (IDO) and arginase during human aging be targets for immunosenescence control?

**DOI:** 10.1186/s12979-021-00244-x

**Published:** 2021-08-13

**Authors:** Ismael Dale Cotrim Guerreiro da Silva, Dirce Maria Lobo Marchioni, Antonio Augusto Ferreira Carioca, Valquiria Bueno, Gisele Wally Braga Colleoni

**Affiliations:** 1 Departament of Gynecology, Escola Paulista de Medicina, Federal University of São Paulo, São Paulo, Brazil; 2 Nutrition Department, School of Public Health, University of São Paulo School of Medicine (FMUSP), São Paulo, Brazil; 3 Nutrition Department, Universidade de Fortaleza (UNIFOR), Fortaleza, Brazil; 4 Department of Microbiology, Immunology and Parasitology, Escola Paulista de Medicina, Federal University of São Paulo, São Paulo, Brazil; 5 Department of Clinical and Experimental Oncology, Escola Paulista de Medicina, Federal University of São Paulo, São Paulo, Brazil

**Keywords:** IDO, Arginase, Aging, Senescence, Immune system

## Abstract

**Background:**

This study aimed to identify novel plasma metabolic signatures with possible clinical relevance during the aging process. A biochemical quantitative phenotyping platform, based on targeted electrospray ionization tandem mass spectrometry technology, was used for the identification of any eventual perturbed biochemical pathway by the aging process in prospectively collected peripheral blood plasma from 166 individuals representing the population of São Paulo city, Brazil.

**Results:**

Indoleamine 2,3-dioxygenase (IDO) activity (Kyn/Trp) was significantly elevated with age, and among metabolites most associated with elevations in IDO, one of the strongest correlations was with arginase (Orn/Arg), which could also facilitate the senescence process of the immune system. Hyperactivity of IDO was also found to correlate with increased blood concentrations of medium-chain acylcarnitines, suggesting that deficiencies in beta-oxidation may also be involved in the immunosenescence process. Finally, our study provided evidence that the systemic methylation status was significantly increased and positively correlated to IDO activity.

**Conclusions:**

In the present article, besides identifying elevated IDO activity exhibiting striking parallel association with the aging process, we additionally identified increased arginase activity as an underlying biochemical disturbance closely following elevations in IDO. Our findings support interventions to reduce IDO or arginase activities in an attempt to preserve the functionality of the immune system, including modulation of myeloid-derived suppressor cells (MDSCs), T cells, macrophages, and dendritic cells’ function, in old individuals/patients.

## Introduction

Immunosenescence is the name of a group of complex alterations in both innate and adaptive arms of the immune system associated with the aging process, leading to a progressive loss in the ability to respond to infections and poor immunity after vaccination [1], both associated with a higher mortality rate in old individuals. The rise in its recognition is pertinent and timely, given the increasing average age and the corresponding failure to increase healthy life expectancy [2].

A low pro-inflammatory phenotype - or inflammaging - in association with an adequate anti-inflammatory profile, could allow people to reach advanced age without disabilities. Thus, remodeling the immune responses seems important to reduce age-related degenerative diseases, both inflammatory and/or neoplastic, to achieve successful aging [3].

The diversity of cells, molecules, and pathways involved in the remodeling of the immune system and their ability to influence each other, including the individual variability of the immune response, turns difficult to identify interventions to improve or maintain the immune function in older adults [4]. In recent years, numerous studies on the mechanisms underlying age-related immune decline became the basis for some interventions such as the reduction of the latent/lytic viral load, by vaccination and/or antiviral drugs, to diminish CMV-related immunosenescence [1, 4]. Supporting this theory, evidence suggests that infections and frailty repeatedly cross each other pathophysiological ways and accelerate the aging process in a vicious circle [5].

Although it is a recent concept and still much discussed, immunosenescence/inflammaging duo (representing two sides of the same phenomenon) may not be harmful but may represent that the most successful changes will guarantee healthy longevity. In addition, cumulative data suggest that, without their existence, human longevity would be greatly shortened [6–8].

Inflammaging and metaflammation largely share the same molecular mechanisms, in which metaflammation can be conceptualized as a specific situation of chronic inflammation caused by nutrient excess. The gut microbiota has a central role in both metaflammation and inflammaging due to its ability to release inflammatory products, contribute to circadian rhythms, and crosstalk with other organs and systems. Therefore, Franceschi et al. [6] argue that chronic diseases are not only the result of aging and inflammaging but accelerate the aging process. Also, they propose the use of metabolic biomarkers that are capable of assessing biological versus chronological age in metabolic diseases [6].

Hence, this study aimed to identify novel plasma biomarkers and metabolic signatures with possible relevance during the aging process. A biochemical quantitative phenotyping platform based on targeted electrospray ionization tandem mass spectrometry technology (ESI-MS/MS) was used to aid in the identification of any eventual perturbed biochemical pathway in peripheral blood plasma from our population-based participants.

## Material and methods

### Participants

**Fig. 1:**
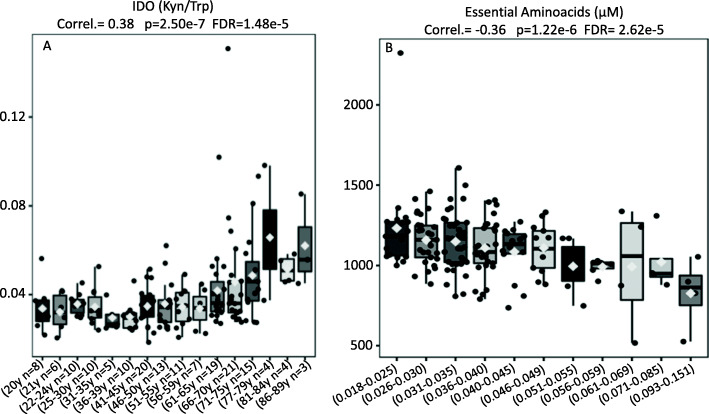
**A** IDO activity (Kyn / Trp) (Y-Axis) is shown to be significantly elevated with age (X-Axis). **B** The sum of the essential amino acids (Y-Axis) shows an inverse correlation with IDO levels (X-Axis)

The study population was selected from the “Health Survey of Sao Paulo (ISA-Capital)” a cross-sectional population-based survey to assess health conditions among a representative sample of individuals living in the City of Sao Paulo, South-eastern Brazil, between 2008 and 2009. A complex probabilistic sampling, by conglomerates, based on census tracts and households that had already drawn in the National Household Sample Survey 2005 (https://www.cps.fgv.br/cps/bd/curso/Household-Surveys-Short-Description-site.pdf) was used. Participants were composed of 166 volunteers (female *n* = 79, male *n* = 87) at ages ranging from 20 to 89 years (mean = 50.7, median = 51) with available, prospectively collected, samples during the accomplishment of the “Health Survey of Sao Paulo (ISA-Capital)”. Due to its population-based characteristics, it was possible to group and analyze systemic metabolic variations of participants, distributed in sixteen progressive subgroups according to increasing ages ranging from 20 to 89 years (Fig. 1A). All plasma samples were collected on at least an 8-h fast in EDTA-containing tubes, centrifuged at 1000-x g for 10 min, transferred to a new tube, and stored immediately at − 80 °C until analysis.

### Metabolomic analysis

Plasma samples stored at − 80 °C were kept on dry ice during transportation. Absolute quantification (μM/L) of peripheral blood metabolites was achieved by targeted quantitative profiling of 186 annotated metabolites by electrospray ionization (ESI) tandem mass spectrometry (MS/MS) in plasma samples, using SCIEX 5500 QTRAPO (SCIEX, Darmstadt, German), blinded to any phenotype information, on a centralized, independent, fee-for-service basis at the quantitative metabolomics platform from BIOCRATES Life Sciences AG, Innsbruck, Austria (https://biocrates.com/) [9, 10].

The experimental metabolomics measurement technique is described in detail by patent US 2007/0004044 (accessible online at http://www.freepatentsonline.com/20070004044.html). Briefly, a targeted profiling scheme was used to quantitatively screen for fully annotated metabolites using multiple reaction monitoring, neutral loss, and precursor ion scans. Quantification of metabolite concentrations and quality control assessment was performed with the MetIQ software package (BIOCRATES Life Sciences AG, Innsbruck, Austria) in conformance with 21CFR (Code of Federal Regulations) Part 11, which implies proof of reproducibility within a given error range. An xls file was then generated, which contained sample identification and 186 metabolite names and concentrations with the unit of μmol/L of plasma (https://biocrates.com/) [9, 10].

In total, 186 annotated metabolites were quantified using the p180 kit (BIOCRATES Life Sciences AG, Innsbruck, Austria), being 40 acylcarnitines (AcylCs), 21 amino acids (AAs), 19 biogenic amines (BA), a sum of hexoses (Hex), 76 phosphatidylcholines (PCs), 14 lysophosphatidylcholines (LPCs) and 15 sphingomyelins (SMs). Glycerophospholipids were further differentiated with respect to the presence of ester (a) and ether (e) bonds in the glycerol moiety, where two letters denote that two glycerol positions are bound to a fatty acid residue (aa = diacyl, ae = acyl-alkyl), while a single letter indicates the presence of a single fatty acid residue (a = acyl or e = alkyl) (https://biocrates.com/) [9, 10].

For metabolomics data analysis, log-transformation was applied to all quantified metabolites to normalize the concentration distributions and uploaded into the web-based analytical pipelines MetaboAnalyst 3.0 (www.metaboanalyst.ca) and Receiver Operating Characteristic Curve Explorer & Tester (ROCCET) available at http://www.roccet.ca/ROCCET for the generation of uni and multivariate Receiver Operating Characteristic (ROC) curves obtained through Support Vector Machine (SVM), Partial Least Squares-Discriminant Analysis (PLS-DA) and Random Forests as well as Logistic Regression Models to calculate Odds Ratios of specific metabolites (http://www.roccet.ca/ROCCET) [9–13].

In addition to individual metabolite quantification, groups of metabolites related to specific functions were assembled as ratios based on the previous observation that the proportions between metabolite concentrations can strengthen the association signal and at the same time provide new information about possible metabolic pathways [14–16]. IDO activity was assessed through Kyn/Trp ratio and arginase activity by Orn/Arg ratio, with or without age correction.

## Results

The indoloxigenase activity, as evaluated by the proportions between kynurenine to the essential amino acid tryptophan (Kyn/Trp), depicted the highest positive correlation with aging (Fig. 1A) (Correl. = 0.38, *p* = 2.50e-7, FDR = 1.48e-5), suggesting that the aging process seems to be driven by deficiencies in auxotrophic-related mechanisms, involving essential amino acids like tryptophan. To further confirm this possibility, we hypothesized that the concentration of essential amino acids should depict an inverse correlation pattern to increases in IDO activity.

**Fig. 2:**
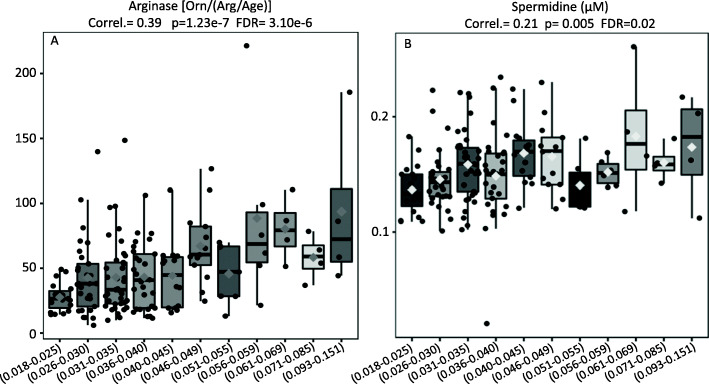
**A** Elevations in arginase activity, evidenced by the proportions of ornithine to age-normalized arginine [Orn/(Arg/Age)] (Y-Axis), were significantly and positively correlated to IDO activity (X-Axis). **B** The molar levels of spermidine (Y-Axis) were significantly correlated to increases in IDO activity (X-Axis)

In agreement with this hypothesis, the molar sum of the 9 essential amino acids (Val, Trp, Thr, Phe, Ile, Leu, Met, Lys, and His) exhibited an opposite and significant correlation with IDO activity (Correl. = − 0.36, *p* = 1.22e-6, FDR = 2.62e-5), showing that nutritional aspects seem to matter in this group of individuals (Fig. 1B). Another important mechanism related to auxotrophy was identified in the present study: elevations in arginase activity, as evidenced by the proportions of ornithine to age-normalized arginine [Orn/(Arg/Age)], were significantly and positively correlated to IDO activity (Fig. 2A).

To further validate these results and knowing beforehand that the activation of arginase and IDO are capable to trigger spermidine production, we postulated that the levels of this biogenic amine should also be elevated. In total support of our hypothesis, the molar levels of spermidine were significantly correlated to increases in IDO activity (Fig. 2B).

**Fig. 3:**
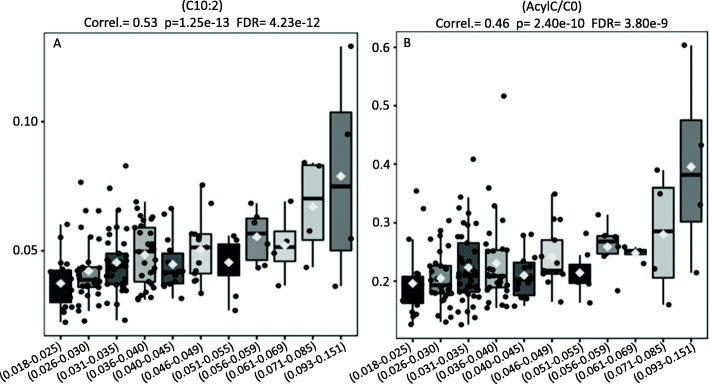
**A** Elevations in the levels of medium-chain acylcarnitines such as C10: 2 (Y-Axis) are associated with elevations in the IDO (X-Axis). **B** Ratio between the total amounts of acylcarnitines (AcylC) to the levels of free carnitine (CO (AcylC/CO) revealed that blood acylcarnitines (AcylC) concentrations are disproportionally elevated concerning free carnitine (CO), suggesting that beta-oxidation deficiencies are associated with increased values in IDO activity (X-axis)

Hyperactivity of indoloxigenase was also found to correlate with increased blood concentrations of medium-chain acylcarnitines, such as decadienylcarnitine (C10:2); this finding suggests that deficiencies in beta-oxidation may also be involved in the immunosenescence process (Fig. 3A) (Correl. = 0.53, *p* = 1.25e-13, FDR = 4.23e-12). To confirm this possibility, we assembled the ratio between the total amounts of acylcarnitines (AcylC) to the levels of free carnitine (CO). In complete alignment with our hypothesis, the increased values generated by the ratio (AcylC/CO) revealed that blood acylcarnitines (AcylC) concentrations are disproportionally elevated concerning free carnitine (CO). As such, these biochemical deviations are highly suggestive that beta-oxidation deficiencies are, indeed, associated to increased values in IDO activity (Fig. 3B) (Correl. = 0.46, *p* = 2.40e-10, FDR = 3.80e-9).

**Fig. 4:**
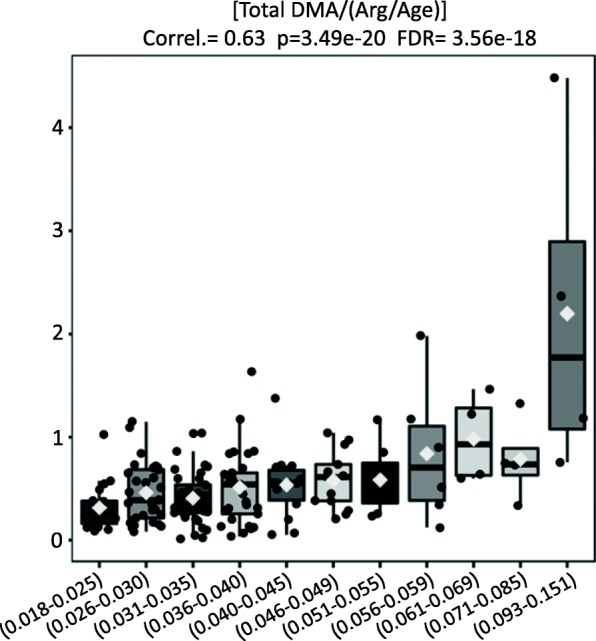
The proportions between the systemic levels of Total Dimethylated Arginine (Total DMA) and unmodified Arginine (corrected for age) (Y-Axis) shows a marked positive correlation with the elevations of IDO (X-axis)

Finally, our study provided evidence that the systemic methylation status was found significantly increased and positively correlated to IDO activity (Correl. = 0.63, *p* = 3.49e-20, FDR = 3.56e-18) as evaluated by the proportions between the molar concentrations of Total Dimethylated Arginine (Total DMA) to its unmodified arginine values (corrected for age) (Fig. 4).

## Discussion

Some amino acids catabolic pathways, as well as normal mitochondrial function, have become critical checkpoints in immunity [17–19]. The necessity for an external source of a nutrient is called auxotrophy. Amino acid auxotrophy is known nowadays to constitute a fundamental immunoregulatory control node that modulates immune responses mostly by tempering T cell activity through a diversity of mechanisms including amino acid starvation [18]. From the amino acids perspective, myeloid-derived suppressor cells (MDSCs) mediate their suppressive activity by promoting the production of arginase and IDO, leading to deprivation of amino acids required for immune cell activation and proliferation [17]. Many amino acids have been found to play important roles as immune regulatory players, including ornithine (Orn), tryptophan (Trp), kynurenine (Kyn), and arginine (Arg) [18]. Therefore, the associated immunoregulatory effects rely on the depletion of specific amino acids in the microenvironment and/or generation of biologically active metabolites [20].

In the present article, besides identifying elevated IDO (Kyn/Trp) activity, exhibiting striking parallel association to the aging processes, we additionally identified increased arginase (Orn/Arg) activity as an underlying biochemical disturbance closely following elevations in IDO. Indeed, the sequential activation of arginase and IDO, promotes a potent immunoregulatory phenotype in which spermidine, a polyamine produced downstream of the arginase-dependent pathway, is capable of triggering IDO phosphorylation and signaling, representing the critical molecular interconnection between the two enzymes [20]. Of note, enzymes that hydrolyze L-tryptophan and that degrade L-arginine are substantially increased in cancer [21], highlighting the potential of these two pathways/enzyme inhibitors in improving immune response in cancer and other immunosuppressive conditions, and contributing to a healthy aging.

The enzyme IDO is responsible for the rate-limiting step of tryptophan catabolism. Metabolites generated from tryptophan catabolism are immunoregulatory molecules, collectively called kynurenines, playing important roles in modulating immune cell function [20]. The role of tryptophan metabolism in immune function has recently become an area of intense study as it relates to the propensity of tumor cells to evade immune cell responses. Enforced expression of IDO in tumor cells impaired the antitumor immune responses by T cells, but this could be overcome by pharmacological inhibition of IDO with 1-methyltryptophan [22, 23]. Therefore, amino acid metabolism in immune cells could yield important new insights into immune cell function [24]. Reinforcing that IDO activity might be a mechanism involved in the decline of T cell responses in immunosenescence, Pertovaara et al. [25] measured Kyn/Trp, reflecting IDO activity, in 284 nonagenarians and 309 blood donor controls. IDO activity was significantly higher in nonagenarians compared with young controls and predicted subsequent mortality in the group of old individuals.

From the standpoint of energy production and mitochondrial function, recent studies have shown that different immune cell subtypes use distinct metabolic programs to perform their functions [19]. Indeed, effector T cells prioritize aerobic glycolysis during anabolic metabolism to balance the synthesis of macromolecules and the generation of energy to support it. Conversely, memory T cells, as well as regulatory T cells (Treg), prioritize fatty acid oxidation (FAO also called beta-oxidation) to support the energy demand for survival and function [26]. Of note, in the present article, we identified disturbances in beta-oxidation as another underlying condition depicting significant correlations to IDO activity. Indeed, the elevated proportions between the sum of esterified carnitines (AcylC) to free carnitine (C0) significantly parallels the increasing levels in IDO activity (Fig. 3B).

L-Arginine is a nonessential amino acid metabolized by arginase 1(Arg1), arginase 2, and inducible nitric oxide (NO) synthase. Arg1 and arginase 2 hydrolyze L-Arginine into urea and L-ornithine, the latter being the main substrate for the production of polyamines that are required for cell cycle progression. Therefore, the accumulation of ornithine and polyamines facilitates cancer cell proliferation. Besides its fundamental role in the hepatic urea cycle, arginase is also expressed in the immune system of mice and men [27].

L-Arginine is also metabolized by inducible NO synthase to citrulline and NO, a highly reactive compound important in vascular homeostasis, and as part of the cytotoxic mechanism of macrophages [28]. Therefore, arginine metabolism has been shown to play a key role in the inflammatory function of macrophages [29]. Macrophages use arginine in two distinct metabolic pathways, the NO synthesis, and the arginase pathways. The pathway used for arginine metabolism in macrophages has profound effects on the immune function of the cell [24]. Macrophage flux of arginine into the NO synthesis pathway is associated with an inflammatory M1 phenotype. In contrast, arginine flux through the arginase pathway is associated with a more tolerant immune response [24]. Difluoromethylornithine, an inhibitor of ornithine decarboxylase has demonstrated promising antitumor effects in mouse models of prostate cancer [30]. Therefore, a reduction in L-arginine may be related to immunosuppression associated with cancer and aging and could be supplemented in an attempt to reverse this deviation [27]. Arginase is also constitutively expressed by human polymorphonuclear granulocytes (PMN) and severely impairs key functions of primary human NK cells as well as IL-2-activated NK cells. In the absence of arginine, NK cell proliferation and IL-12/IL-18-induced secretion of IFN-gamma are severely diminished [31].

Therefore, Arg1 and IDO1 are immunosuppressive enzymes known to operate in distinct immune cells. Mondanelli et al. [32] demonstrate that Arg1 and IDO1 cooperate in conferring long-term, immunosuppressive effects on dendritic cells and exert proapoptotic and antiproliferative effects on T-cells, either by a shared, well-described molecular pathway, but also by other separate mechanisms [33].

Arginine methylation is known to play a major role in gene regulation because of the ability of the predominant methyltransferase (PRMTs) to deposit key activating (histone H4R3me2a, H3R2me2s, H3R17me2a, H3R26me2a) or repressive (H3R2me2a, H3R8me2a, H3R8me2s, H4R3me2s) histone marks. In addition, there are many substrates that are non-histones involved in biological processes including transcription, cell signaling, mRNA translation, DNA damage signaling, receptor trafficking, protein stability, and pre-mRNA splicing [34, 35]. Therefore, arginine methylation has emerged as a key regulator of signal transduction with an important role in T lymphocyte activation. The PRMT-1 is highly expressed in T helper cells, and ligation of the T cell antigen and costimulatory receptors induces arginine methylation on several cytoplasmic proteins. Global inhibition of methyltransferases can result in signaling defects in CD4+ T cells and profound immunosuppression. Here we suggest that manipulating arginine methylation could be a feasible strategy to modulate T lymphocyte function, presenting a novel approach towards immunotherapy and the treatment of T cell-mediated disorders such as autoimmune disease and transplant rejection [36].

Besides IDO1 and Arg 1, other amino acid degrading enzymes such as tryptophan 2,3-dioxygenase and arginase 2, has relevance in the regulation of tumor-induced immune tolerance, including the induction of an immunosuppressive tumor microenvironment [23]. IDO1-mediated tryptophan degradation can promote Treg cell differentiation and activation, which, in turn, leads to the expansion and intratumoral recruitment of Arg-competent immunosuppressive myeloid populations like myeloid-derived suppressor cells (MDSCs). Preclinical data suggest that IDO1 inhibition by INCB024360 will increase T cell proliferation, decrease Treg cells and MDSCs activity. In Phase II clinical trial in myelodysplastic syndrome patients, although the mean kynurenine/tryptophan ratio decreased 42% at cycle 2, the alterations in MDSCs and T effector cells were not significant and 80% of patients achieved only stable disease as the best response [37].

MDSCs significantly increase with aging and are the enhancers of other immunosuppressive cells, such as Treg and regulatory B cells (Bregs). Therefore, one could think that MDSCs remodel the immune system, preventing excessive inflammation with aging and inducing immunosenescence [38].

MDSCs cause suppression of immune cells via several mechanisms, notably through reactive oxygen species (ROS) generated by g-MDSCs, NO generated by m-MDSCs, production of arginase, and also by secretion of cytokines such as IL-10 and TGF-β. Arginine is used metabolically by MDSC and thus by removing this substrate from the microenvironment that is also used by T cells, MDSC prevents its proliferation [39]. MDSCs can then further inhibit antitumor effector T-cell responses through arginase-, iNOS-, PD-L1- and inhibitory cytokine-dependent mechanisms [23]. Thus, the consumption of L-arginine by Arg 1 represents a well-known immunoregulatory mechanism exploited by M2 macrophages and MDSCs [20].

**Fig. 5:**
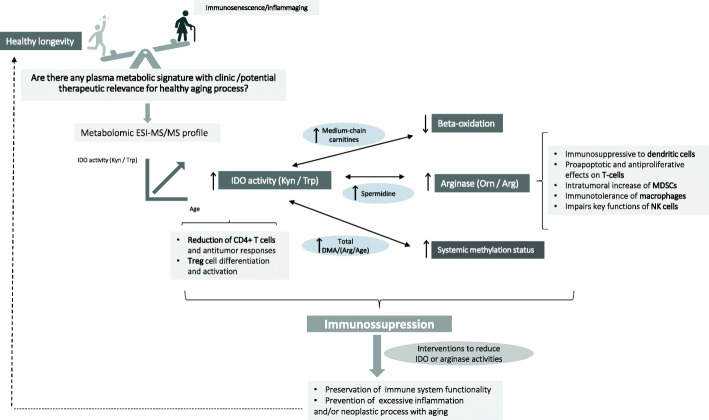
Study overview and literature revision, as described in the discussion section

Observed age-associated dysfunction of macrophages is the result of their functional adaptation to the age-associated changes in tissue environments. The macrophages appear to maintain functional plasticity during this dysregulation, making them a prime target of cytokine therapy that could enhance both innate and adaptive immune systems [40]. In cancer, however, both ARG1 and IDO1 are overexpressed, in MDSCs and DCs, and contribute to the impairment of the host anti-tumor immunity [20]. Therefore, is ARG1 inhibition a good therapeutic strategy? The use of arginase inhibitors such as nor-N-hydroxy-L-arginine abrogated the arresting effects of arginase on T-cell proliferation and allowed lymphocyte-dependent tumor reduction [28]. As MDSCs are also able to polarize macrophages toward the M2 phenotype, arginase inhibitors could have a double effect in favor of T cells proliferation and preventing the expansion of immunosuppressive tumor-associated macrophages (TAMs) [41]. Further investigations on arginine metabolism in the aging process, neoplasia, and also autoimmune disorders, and its possible relationship with IDO1 is a new pathway to be explored, with possible therapeutic purposes (Fig. 5).

One limitation of this study is to hypothesize that metabolomic findings related to aging are also related to immunosenescence. However, the correlations are entirely theoretical and based on literature data. Future studies with immune subpopulations could reinforce the present hypothesis.

In conclusion, in the present article, besides identifying elevated IDO activity exhibiting striking parallel association with the aging process, we additionally identified increased arginase activity as an underlying biochemical disturbance closely following elevations in IDO. Our findings support interventions to reduce IDO or arginase activities in an attempt to preserve the functionality of the immune system, including modulation of MDSCs, T cells, macrophages, and dendritic cells’ function, in old individuals/patients.

## Data Availability

Our raw data was deposit in Metabolights (www.ebi.ac.uk/metabolights/MTBLS2260).

## Abbreviations

AAs: Amino acids
AcylC: Acylcarnitines
Arg1: Arginase 1
BA: Biogenic amines
Breg: Regulatory B cells
C10:2: Decadienylcarnitine
CO: Free carnitine
EDTA: Ethylenediaminetetraacetic acid
ESI-MS: Electrospray ionization tandem mass spectrometry
FAO: Fatty acid oxidation or β-oxidation
FDR: False discovery rates
Hex: Sum of hexoses
IDO: Indoleamine 2,3-dioxygenase
ISA-Capital: Health Survey of Sao Paulo
Kyn/Trp: Kynurenine to tryptophan ratio
LPCs: Lyso-phosphatidylcholines
MCCV: Monte-Carlo Cross Validation
MDSCs: Myeloid-derived suppressor cells
MS: Mass spectrometry
NO: Nitric oxide
Orn/Arg: Ornithine to arginine ratio
PCs: Phosphatidylcholines
PLS-DA: Partial Least Squares-Discriminant Analysis
PMN: Polymorphonuclear granulocytes
PRMTs: Predominant methyl transferase
ROC: Receiver Operating Characteristic
ROS: Reactive oxygen species
SMs: Sphingomyelins
SVM: Support Vector Machine
TAMs: Tumor associated macrophages
Treg: Regulatory T cells
